# Mineralogical and radiological studies on some Paleozoic yellow ochre deposits in Southwestern Sinai, Egypt

**DOI:** 10.1038/s41598-024-72735-y

**Published:** 2024-10-02

**Authors:** Marwa M. Abdel-Azeem, Nareman M. Harpy, Abdalla S. Alshami, Wael M. El Maadawy

**Affiliations:** Nuclear Materials Authority, El Maadi, P.O. Box 530, Cairo, Egypt

**Keywords:** Yellow ochre, Paleozoic sedimentary rocks, Radioactivity levels, Radiation hazard, Environmental assessment, Environmental sciences, Natural hazards

## Abstract

Yellow ochre is the basic material used in the manufacture of yellow oxide (a commercial product). Yellow ochre samples were taken from three different formations in southwestern Sinai: Abu Hamata, Um Bogma, and Abu Zarab. Yellow ochre occasionally exists in Abu Hamata Formation particularly in El Ferah area, associated with Fe–Mn ore in Wadi El Sahu (Um Bogma Formation) and in Himayer area (Abu Zarab Formation). The XRD analysis of the raw material reveals that they are mainly composed of goethite, which is associated with quartz and kaolinite in El Ferah area, hematite, kaolinite and quartz in Himayer area, and kaolinite, gypsum and quartz in Wadi El Sahu. The commercial product is mainly composed of goethite, quartz, and calcite. The heavy mineral investigation shows that some yellow ochre samples contain zircon and rare earth sulfate which may be responsible for the radioactivity of ochre due to their thorium and uranium content. The average values of specific radio-activities of most radionuclides in the samples of Himayer area I and II, and El Sahu I are higher than the respective world averages, while their activities are lower in El Ferah and El Sahu II. Th/U and Ra/U ratios exhibit vigorous changes in physico-chemical conditions during uranium leaching and deposition. Most of the radiological parameters in the ferruginous sediment and commercial product samples from El Ferah, El Sahu II, and Himayer II are lower than the recommended international values but higher than those from Himayer I and El Sahu I samples. The plotted hierarchical cluster analysis (HCA) exhibits that the main contributors for the hazards of these sediments and their commercial product are ^238^U, ^232^Th and ^226^Ra in Himayer I &II, commercial products, and El Ferah area, ^232^Th and ^226^Ra in El Sahu II , ^232^Th, ^40^K and ^226^Ra in El Sahu I.

## Introduction

Naturally occurring radioactive materials (NORM) refer to natural radionuclides, predominantly ^238^U, ^232^Th, ^226^Ra, ^40^K and others, which arise from natural processes rather than human activities. They are ubiquitously present in various components of the natural environment, such as rocks, soil, water, and atmosphere, as well as flora and fauna^1,2^. Many individuals may be unaware of the inherent presence of natural radioactivity in our environment, as it is widespread and can be detected in rocks, soil, and air-essentially omnipresent^3^. The specific activities are related to and correlated with these elements, chemical, and geological properties of the soil. Leaching, surface erosion and plant uptake are also major factors affecting the distribution of radionuclides^4–6^.

The impact of radiation on the public is a focal point in radio-ecological research. Numerous studies have been conducted to gauge the radiological risks and the annual dosage attributed to natural radioactivity within buildings. Conducting a thorough radiological impact assessment of building materials is of paramount importance to both estimate and manage the potential effects on public health and the environment. This undertaking is particularly crucial and delicate, given the stringent criteria of sustainable development.

Indoor ornamental materials such as rocks and pigments can improve the appearance and atmosphere of a building^7^. Because of their inherent radionuclide concentration, building materials can provide considerable gamma dose inside^8^, and contribute to radon exposure as well^9^. High activity concentrations of radionuclides in building materials may increase interior gamma radiation exposure and external dose rates. Furthermore, if these materials are derived from rock and soil, indoor radiation exposure may be higher than outdoor exposure^10^. The International Commission on Radiological Protection has discovered significant disparities in gamma radiation exposure between populations living in sedimentary and granite environments. Nonetheless, because individuals spend around 80% of their time inside, they may be exposed to radioactive dangers from these construction materials^11^.

Chronic exposure of humans to modest amounts of ionizing radiation can create health problems 5–30 years after the exposure. The most serious consequence of exposure is an increase in the likelihood of the person and his progeny getting malignant illnesses. The danger increases with the dose, and the likelihood of harm appearing is higher when exposure begins at a younger age^12^.

Ochre is mostly utilized in the production of wall pigments and dyes. The name ‘ochre’ widely refers to earth pigments ranging in color from red to brown to yellow to purple. Most often, these pigments are various kinds of iron oxide minerals that provide their color qualities^13^. Iron oxides are the phases responsible for ochre color, with hematite Fe_2_O_3_ and goethite (FeO(OH)) forming iron as the major chromophore for red and yellow, respectively^14^.

Yellow ochre (used in the production of yellow oxide pigment) is generated from the mineral goethite FeO (OH), the chemical composition of goethite is as follows: Fe (63%), O (27%), and H_2_O (10%), with Mn sometimes present in levels up to (5%), and is most usually coupled with other iron oxides such as limonite and hematite. Weathering or hydrothermal alteration of iron-bearing minerals under oxidizing environments produces goethite^15,16^. It is also found as a deposit in bogs and springs as a direct inorganic or biogenic precipitate from water^15^. Ochre may occur in sedimentary, metamorphic, and igneous environments, although it is most commonly seen in worn sedimentary settings^17^.

Yellow ochre, primarily composed of rock, contains natural radioactive isotopes from the ^232^Th and ^238^U decay series, as well as ^40^K. The gamma radiation emanating from these naturally occurring radioisotopes collectively referred to as terrestrial background radiation, exists in minute concentrations within all ground formations.

Yellow ochre exists in different rocks of Paleozoic formations in southwestern Sinai; especially Abu Hamata, Um Bogma, and Abu Zarab formations. The Paleozoic formations were studied by several authors due to their high contents of the radioactive, trace-, REEs- and heavy metals- bearing mineralization. Abdel Monem^18^ recorded small lenses of radioactive black sand in the Lower Sandstone Series in Wadi El Seih. The recent studies carried on Abu Hamata siliciclastic show that they contain a large assemblage of U and Th-bearing minerals that have several modes of occurrence, including kasolite, uranothorite, zircon, monazite, allanite, and xenotime^19^.

Um Bogma Formation, also represents a radioactive anomaly, El Sharkawi^20^ paid high attention to the lower member of Um Bogma Formation and they regarded it as karst profile. El Aassy^21^ concluded that uranium in the Paleozoic rocks in Um Hamd and Ramlet Hemiyir areas is adsorbed on clays and ferromanganese materials. Shata and Mira^22^ reported that the black carbonaceous shales in Um Bogma area host a large budget of uranium and LREEs-bearing minerals (such as bastnäsite, torbernite), together with some heavy metals.

In contrast, all the previous studies show that Abu Zarab Formation represents the least radioactive rock unit among the seven formations constituting the Paleozoic succession.

This study focuses on investigating the environmental impact of iron oxides and related products, specifically regarding the adsorption of uranium and other radioelements. The research centers on yellow ochre, both as a raw material and in the form of representative yellow oxide samples used as indoor ornamental materials. Concentrations of natural radionuclides, including ^238^U, ^226^Ra, ^232^Th, and ^40^K, were measured in yellow ochre samples collected from southwestern Sinai. Mineralogical investigations will be conducted to identify the radioactive minerals responsible for potential radiological hazards associated with these materials. The assessment of radiological hazards aims to provide crucial information about potential risks to human health arising from the use of these materials in buildings, particularly in domestic interior decorations.

### Analytical techniques

**Fig. 1:**
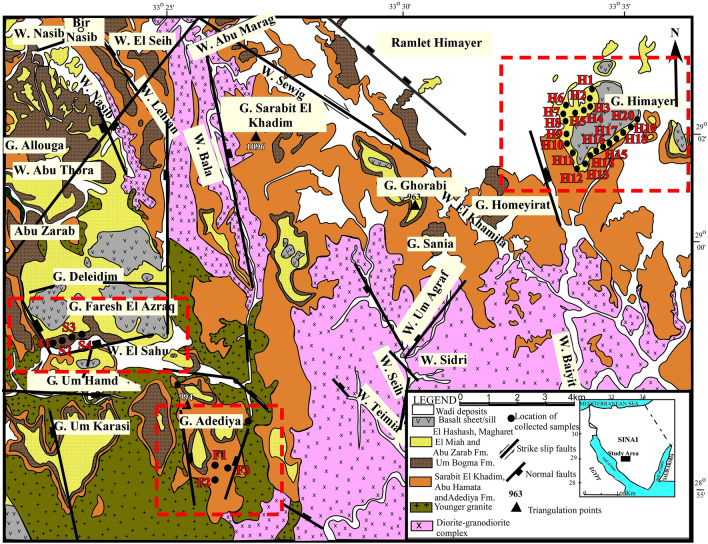
Geologic map of the studied areas in southwestern Sinai. (compiled after^23,24^).

A total of (35) yellow ochre samples (raw material) were collected from southwestern Sinai, (3) samples from El Ferah area, (6) samples were collected from Wadi El Sahu area, and (25) samples were collected from Himayer area (Fig. 1). In addition, (18) representative yellow oxide samples (commercial product) derived from the raw material were bought from different companies and subjected to various analytical techniques.

To produce a homogenous particle size, the yellow ochre samples were crushed into a fine powder with a mixer Mill type MM-2 and sieved through a 2 mm sieve. To remove moisture from the samples, they were all dried in an oven at 110^o^ C for 24 h. Bromoform (specific gravity = 2.89 g/cm^3^) was used in heavy mineral separation from the sand size fractions (2 to 0.063 mm) in the obtained ochre samples. The chemical composition of the separated mineral grains was identified using an XL30 Phillips scanning electron microscope (SEM) equipped with an energy dispersive X-ray micro-analyzer (EDX). The mineralogical composition of both the bulk yellow ochre and yellow oxide samples was determined using X-ray diffraction (XRD).

The yellow ochre and yellow oxide samples were processed for radioactivity measurements, with a focus on elements such as uranium (^238^U), thorium (^232^Th), radium (^226^Ra), and potassium (^40^K). Each sample was crushed, ground to a particle size of about 1mm, and put in a sealed cylindrical plastic container (9.5 cm diameter, 3cm height) containing approximately 200ml of the sample. The containers were sealed for 28 days to enable the accumulation of free radon and reach radioactive equilibrium.

For measurements, a bicorn-scintillation detector with NaI (Tl), sized 76 × 76 mm, along with an amplifier model NE-4658 and a high voltage power supply model TC-952, was utilized. The instrument setup included a Nucleus PCA-8000 computer-based 8192 multichannel analyzer with a color graphical display and an Epson LX-80 printer. Each sample underwent two measurements, each lasting 1000 s, and the average of the gross counts was calculated for analysis.

The measurements of ^238^U, ^232^Th, ^226^Ra, and ^40^K were conducted using four energy regions at specific keV values: 93 keV, 239 keV, 352 keV, and 1460 keV for ^238^U, ^232^Th, ^226^Ra, and ^40^K, respectively.

The investigated samples were compared to International Atomic Energy Agency (IAEA^25^) standards, namely RGU-1, RGTh-1, and RGK-1, provided by the IAEA for uranium (U), thorium (Th), and potassium (K). The use of these standards allows for a reference point and helps ensure the accuracy and reliability of the measurements.

### Geologic setting of yellow ochre samples

**Fig. 2:**
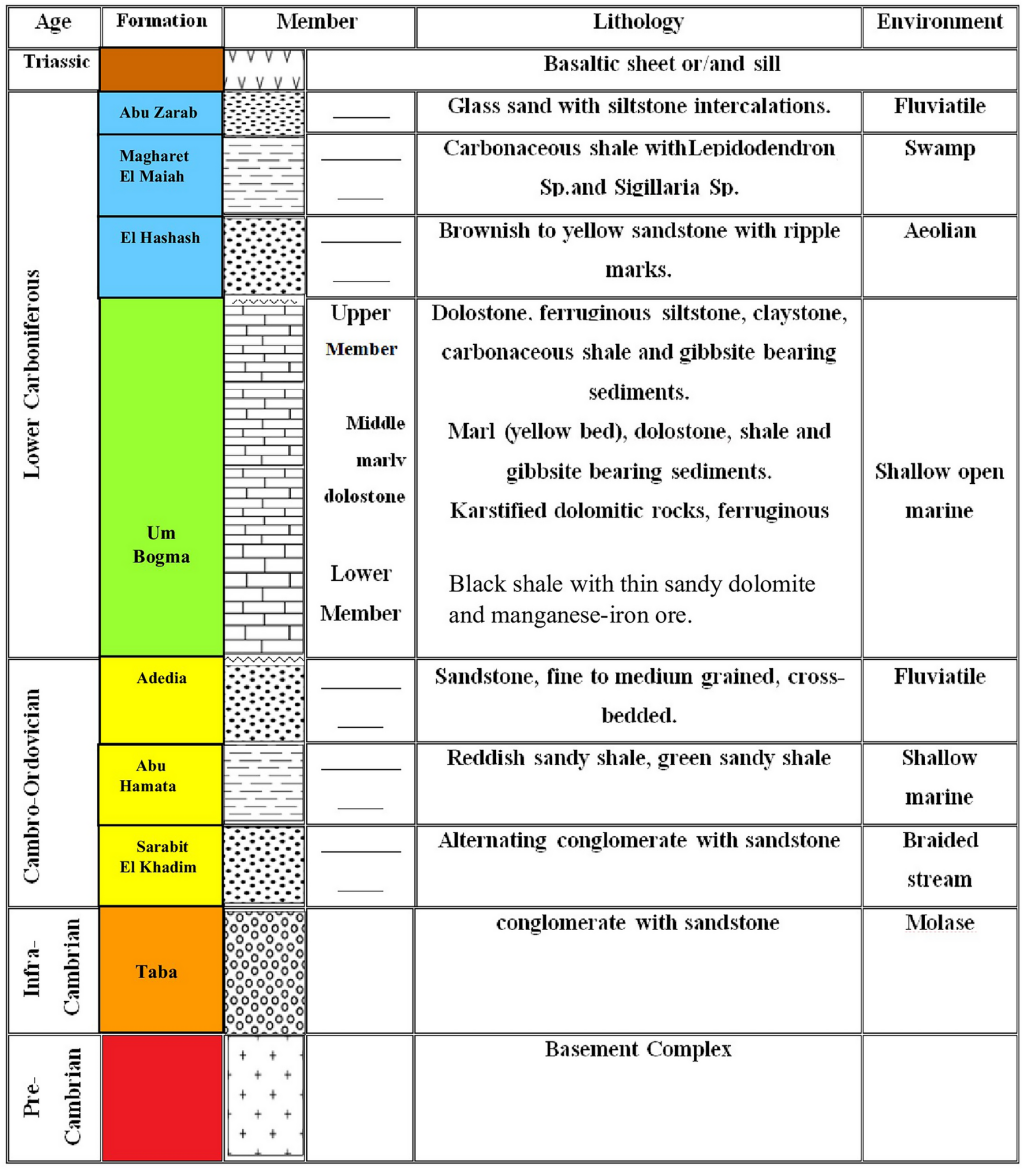
Composite lithostratigraphic section of southwestern Sinai (modified after Alshami^27^). Not to scale.

Southwestern Sinai is mostly covered by Precambrian Basement Complex that is nonconformably overlain by the Paleozoic succession (up to 450 m thick) that is separated from the overlaying Precambrian succession in certain places by basaltic sheets. Weissbroad^26^ divided this Paleozoic succession in south Sinai into: Sarabit El-Khadim, Abu Hamata, Adediya, Um Bogma, El Hashash, Magharet El Miah and Abu Zarab formations (from the oldest to the youngest) (Fig. 2). Yellow ochre is gathered in this study from three formations in southwestern Sinai: Abu Hamata, Um Bogma, and Abu Zarab (Fig. 2). The geologic context of the three formations and sampling locations are described briefly below.

#### Abu Hamata Formation

Abu Hamata Formation conformably overlies Sarabit El Khadim Formation. EL Shahat^28^ suggested a probable Cambro-Ordovician age for the whole lower sandstone series.

**Fig. 3:**
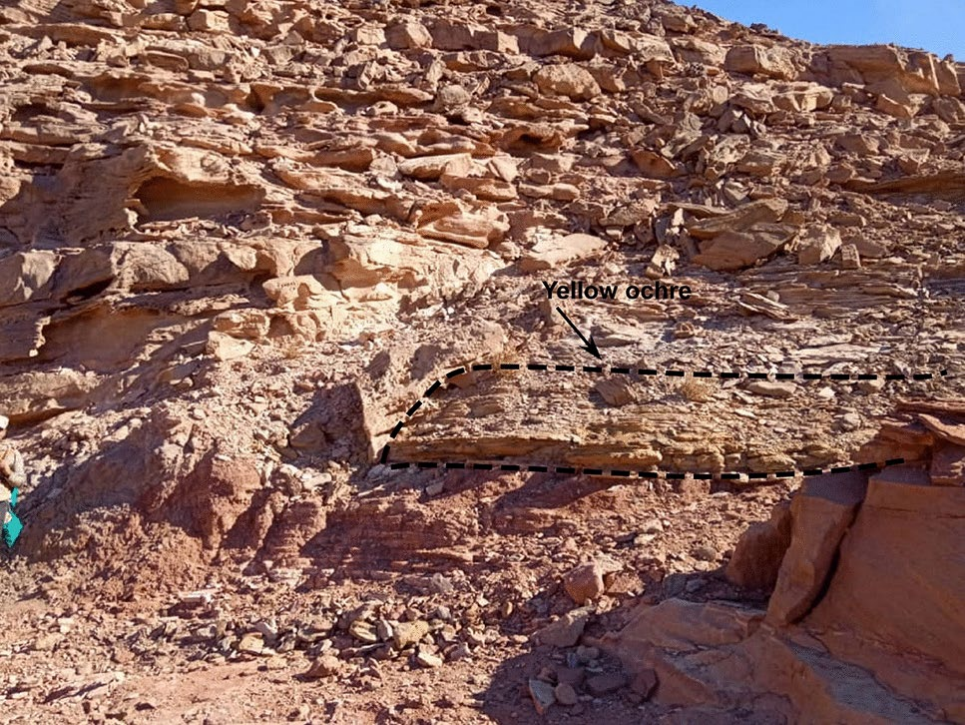
Field photograph showing the yellow ochre of Abu Hamata Formation (outlined) in the El Ferah area (looking south).

The formation is represented by grey to dark grey fine-grained sandstone at its lower part and pale green and very fine-grained sandstone to siltstone in the upper part^19^. This formation extends for tens kilometers and recognized in the field by its distinctive green color. The grey siltstone contains copper mineralization and manganese dendrites^20^. Yellow ochre occasionally exists in Abu Hamata Formation, particularly in El Ferah area (Fig. 3), which is located at the intersection of longitude 33° 25′ 30’’ E and latitude 28° 57′ 33’’ N.

#### Um Bogma Formation

The Um Bogma Formation has unconformable relationships with the underlying and overlying formations^29,30^. Based on its fossil content, most authors agreed that this formation is Early Carboniferous in age. The formation has a maximum thickness of 60 m in its type locality (W. Khaboba at east Abu Zenima area) in southwestern Sinai^31,32^ and is formed mostly of argillaceous rocks, sandstones, carbonates, and ironstone. It was first subdivided by Omara and Schultz^33^ into three members; the lower dolomitic member, the middle dolomitic limestone, marl member, and the upper dolomitic member. Alshami^32^ reported that Um Bogma Formation comprises of seven facies, these facies are Fe–Mn ore, gibbsite-bearing sediments, claystone, marl, dolostone, shale, and ferruginous siltstone. This study focuses on the lower dolostone member which is the oldest rock of the Carboniferous age and is consisted of black shale with thin sandy dolomite and manganese–iron ore^34^. 

**Fig. 4:**
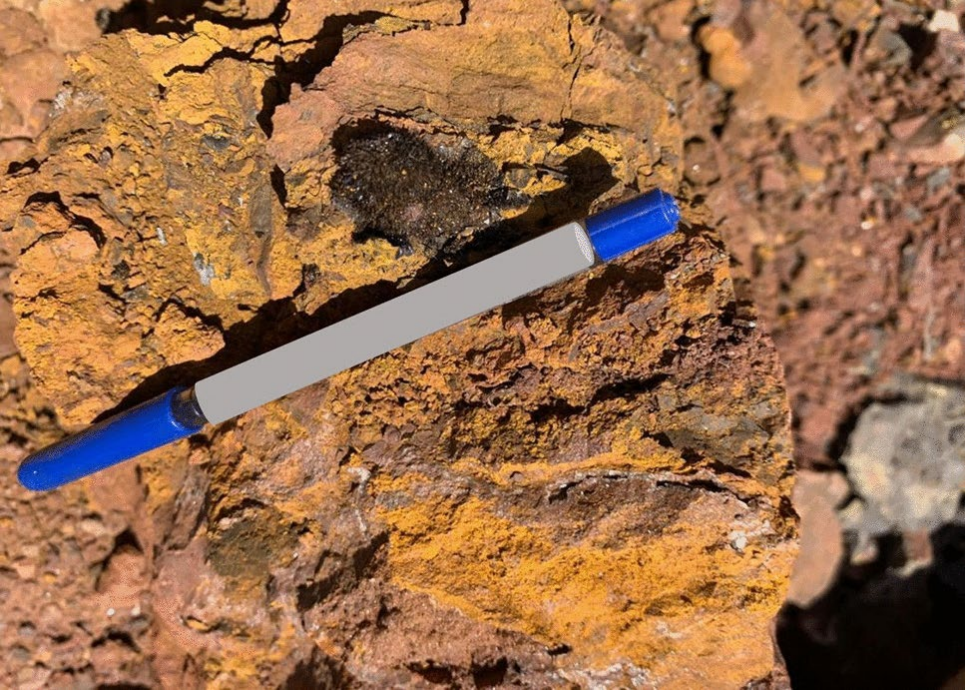
Field photograph showing the yellow ochre of Um Bogma Formation in El Sahu area.

The yellow ochre is associated with Fe–Mn ore, especially in Wadi El Sahu (Fig. 4). Wadi El Sahu area is located at the intersection of longitude 33° 23′ 55’’ E and latitude 28° 58′ 28’’ N.

#### Abu Zarab Formation

**Fig. 5:**
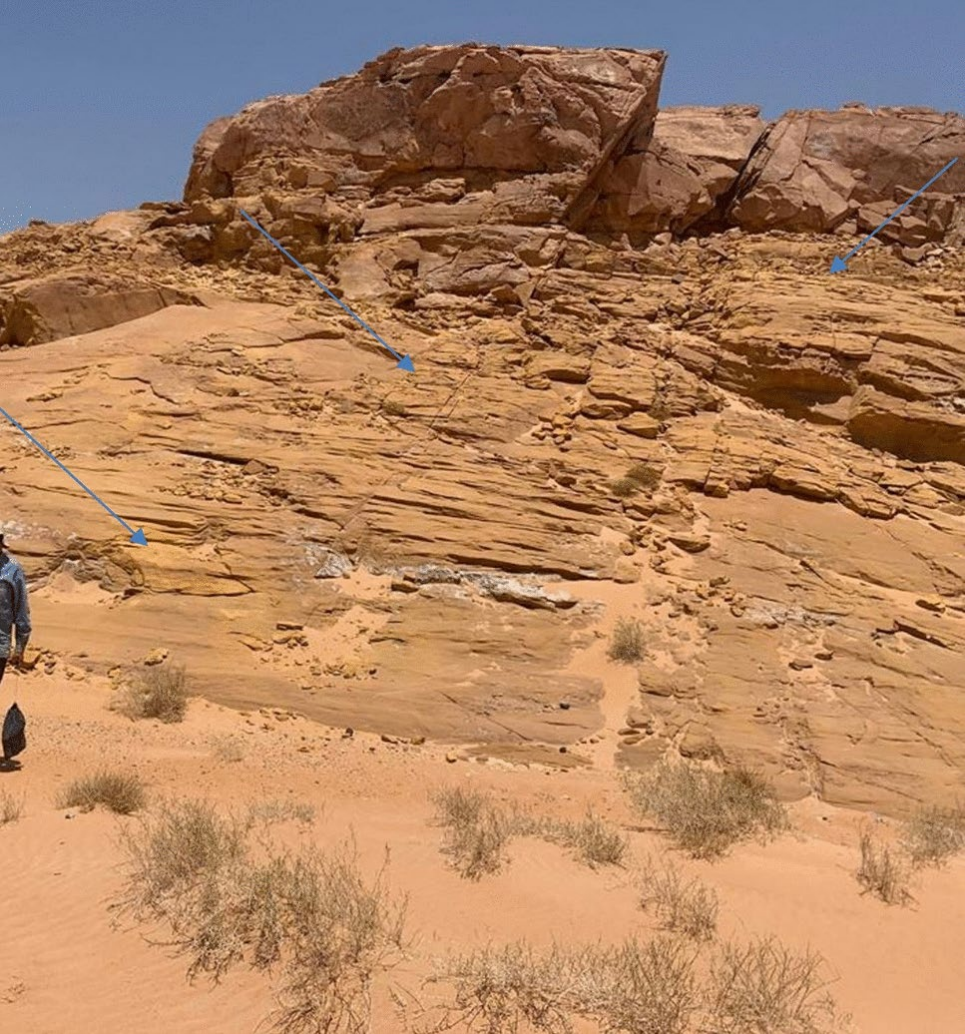
Field photograph showing the yellow ochre of Abu Zarab Formation (arrows) in Himayer area (looking north).

It conformably overlies the Magharet El Miah Formation and acquires a thickness of ~ 26 m at its type locality (Abu Zarab area) and ~ 93 m thick in G. Homiyer^35^. This Formation consists of white, semi-friable, sandstone with siltstone and shale intercalations. Some sandstone beds of this Formation host yellow ochre, its thickness is about 15 m. The yellow ochre in Abu Zarab Formation is recognized in several areas including Himayer and Dabbet El Qeri. In this study, the yellow ochre samples were collected from Himayer area (Fig. 5). G. Himayer extends along a NE—SW trend and is located at the intersection of longitude 33° 33′ 09’’ E and latitude 29° 01′ 36’’ N.

### Mineralogical investigation

**Fig. 6:**
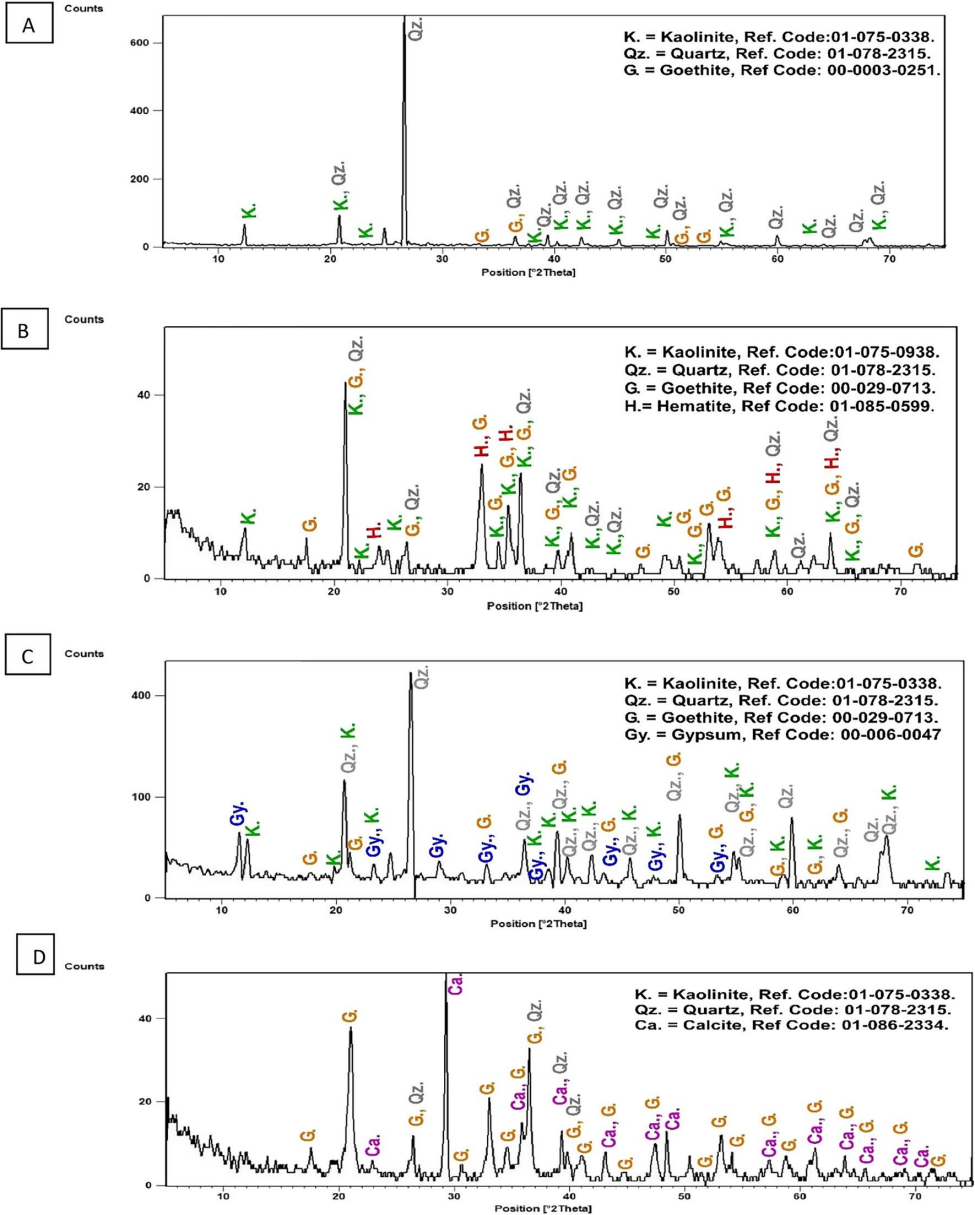
X-ray diffraction pattern of the yellow ochre samples in (**A**) El Ferah area, (**B**) El Sahu area, (**C**) Himayer area, and (**D**) X-ray diffraction pattern of the yellow oxide.

The XRD analysis of the raw material shows that they are mainly composed of goethite mineral which is responsible for the distinctive yellow color. In El Ferah area goethite is associated with quartz and kaolinite (Fig. 6A), in Himayer area with hematite, kaolinite, and quartz (Fig. 6B), while in Wadi El Sahu goethite with kaolinite, gypsum and quartz (Fig. 6C). The XRD pattern for the commercial product shows that it is mainly composed of goethite, quartz and calcite (Fig. 6D).

**Fig. 7:**
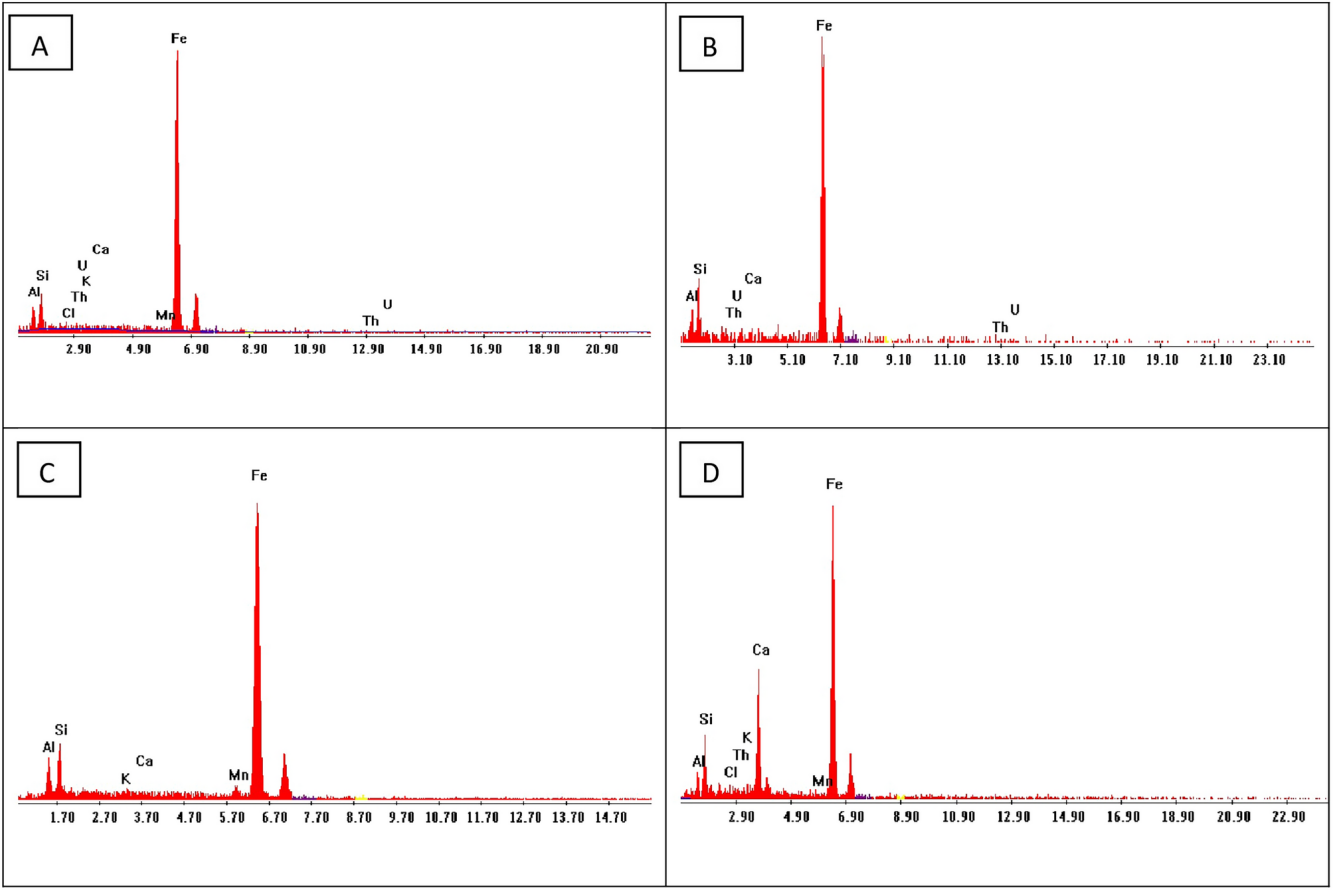
EDX patterns of the yellow ochre samples in: (**A**) El Ferah area, (**B**) El Sahu area, (**C**) Himayer area, and (**D**) EDX pattern of the yellow oxide.

The EDX analyses data (Fig. 7) show that the yellow ochre of El Ferah area is composed of Fe (79.66%), Si (11.37), Al (8.43), with a small amount of Ca (0.58%). While in El Sahu area the yellow ochre consists of Fe (74.37), Si (8.73%), Al (6.48%), Mn (1.18%), Th (3.31%), U (2.72%), K (0.38%), Ca (0.38%) and Cl (0.59%). In addition, in the Himayer area, the yellow ochre is composed of Fe (75.82%), Si (11.31%), Al (9.38), Mn (2.29%), K (0.75%), and Ca (0.44%). The EDX data of the commercial product show that it is mainly composed of Fe (67.14%), Si (10.05%), Al (4.92%), Ca (14.09%), Th (1.7%), Mn (0.84%), K (0.90%), and Cl (0.89%).

**Fig. 8:**
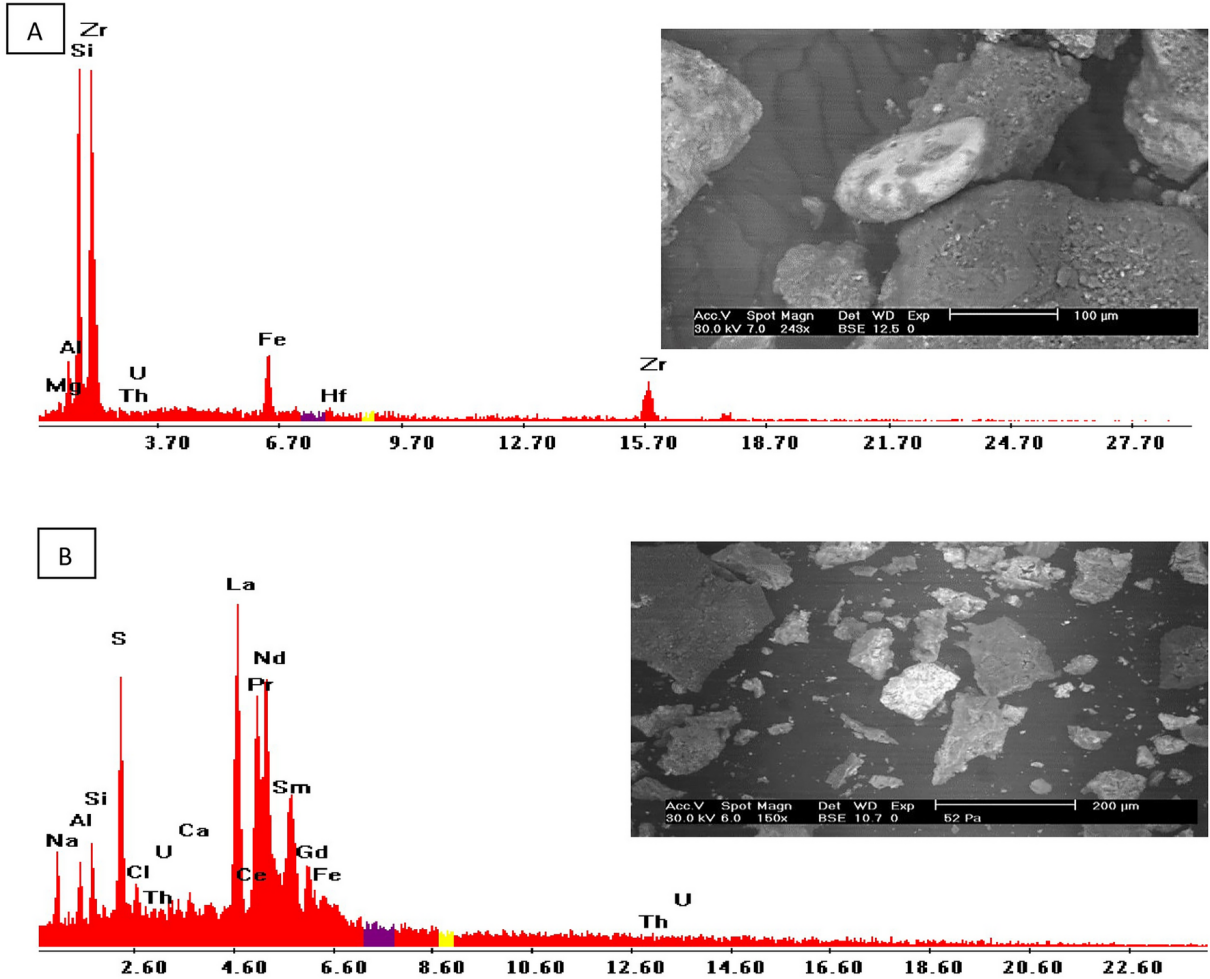
BSE image and EDX pattern of (**A**) Zircon, and (**B**) REE-sulfate.

The heavy mineral investigation shows that some yellow ochre samples contain zircon and rare earth sulfate which may be responsible for their thorium and uranium contents. The EDX data (Fig. 8A) show that zircon mineral is composed of Zr (65.22%), Si (17.77%), Al (3.31%), Hf (2.91%), Fe (7.15%), U (1.05%), and Th (1.03%). The rare earth sulfate (Fig. 8B) consists of S (9.28%), La (29.46%), Ce (0.30%), Pr (6.78%), Nd (24.9%), Sm (3.19%), Gd (1.45%), Fe (1.96%), Th (3.07%), and U (3.24%).

### Specific radioactivity

Radioactive particles, which often contain high concentrations of radioactivity, pose potential risks to human health and the environment. Therefore, their detection, quantification, and characterization are crucial for understanding their impact^36^, the worldwide average activity concentrations for ^238^U, ^226^Ra, ^232^Th, and ^40^K in soils and building materials, as reported by the United Nations Scientific Committee on the Effects of Atomic Radiation (UNSCEAR), are 33, 32, 45, and 412 Bq kg^−1^, respectively. It is important to note that these values can vary regionally based on geological and environmental factors.

The radioactivity data in Table 1, indicate that the average values of the specific activities for ^238^U, ^226^Ra, ^232^Th, and ^40^K in the El Ferah area were 19.10, 15.26, 23.82, and 163.54 Bq kg^−1^, respectively, all below the respective world averages of 33, 32, 45, and 412 Bq kg^−1^as established by UNSCEAR^37^.

**Table 1:** eU, eTh, Ra and K concentrations of the studied areas.

	Sample	eU	eTh	eRa	K	Th/U	Ra/U	^238^U	^232^Th	^226^Ra	^40^K
	No	ppm	ppm	ppm	%			Bq kg^−1^	Bq kg^−1^	Bq kg^−1^	Bq kg^−1^
Himayir I area	H1	1.00	17.00	65.00	0.56	17.00	65.00	12.40	68.68	721.50	175.28
	H2	1.30	23.00	63.00	0.70	17.69	48.46	16.12	92.92	699.30	219.10
	H3	1.50	20.00	68.00	0.34	13.33	45.33	18.60	80.80	754.80	106.42
	H4	1.44	23.00	68.00	1.00	15.97	47.22	17.86	92.92	754.80	313.00
	ave	**1.31**	**20.75**	**66.00**	**0.65**	**16.00**	**51.50**	**16.25**	**83.83**	**732.60**	**203.45**
	min	**1.00**	**17.00**	**63.00**	**0.34**	**13.33**	**45.33**	**12.40**	**68.68**	**699.30**	**106.42**
	max	**1.50**	**23.00**	**68.00**	**1.00**	**17.69**	**65.00**	**18.60**	**92.92**	**754.80**	**313.00**
Himayir II area	H5	1.00	5.00	9.00	2.60	5.00	9.00	12.40	20.20	99.90	813.80
	H6	1.10	8.00	3.00	1.53	7.27	2.73	13.64	32.32	33.30	478.89
	H7	1.33	7.00	4.00	1.11	5.26	3.01	16.49	28.28	44.40	347.43
	H8	1.01	12.00	5.00	1.23	11.88	4.95	12.52	48.48	55.50	384.99
	H9	1.12	8.00	7.00	2.33	7.14	6.25	13.89	32.32	77.70	729.29
	H10	1.08	3.00	5.00	1.57	2.78	4.63	13.39	12.12	55.50	491.41
	H11	1.20	6.00	7.00	2.47	5.00	5.83	14.88	24.24	77.70	773.11
	H12	1.45	5.00	9.00	3.16	3.45	6.21	17.98	20.20	99.90	989.08
	H13	1.04	4.00	6.00	1.30	3.85	5.77	12.90	16.16	66.60	406.90
	H14	1.30	5.00	6.00	1.73	3.85	4.62	16.12	20.20	66.60	541.49
	H15	1.14	3.00	6.00	1.53	2.63	5.26	14.14	12.12	66.60	478.89
	H16	1.09	9.00	8.00	2.27	8.26	7.34	13.52	36.36	88.80	710.51
	H17	1.00	6.00	3.00	0.78	6.00	3.00	12.40	24.24	33.30	244.14
	H18	1.07	5.00	6.00	1.20	4.67	5.61	13.27	20.20	66.60	375.60
	H19	1.45	5.00	10.00	3.12	3.45	6.90	17.98	20.20	111.00	976.56
	H20	1.22	7.00	6.00	3.15	5.74	4.92	15.13	28.28	66.60	985.95
	H21	1.00	4.00	6.00	3.64	4.00	6.00	12.40	16.16	66.60	1139.32
	H22	1.10	1.00	9.00	2.84	0.91	8.18	13.64	4.04	99.90	888.92
	H23	0.95	4.00	6.00	2.24	4.44	6.32	11.16	16.16	66.60	701.12
	H24	0.98	6.00	6.00	2.24	6.12	6.12	12.15	24.24	66.60	701.12
	H25	1.16	3.00	9.00	4.32	2.50	7.76	14.88	12.12	99.90	1352.16
	ave	**1.13**	**5.52**	**6.48**	**2.21**	**4.96**	**5.73**	**14.04**	**22.32**	**71.89**	**690.98**
	min	**0.95**	**1.00**	**3.00**	**0.78**	**0.91**	**2.73**	**11.16**	**4.04**	**33.30**	**244.14**
	max	**1.45**	**12.00**	**10.00**	**4.32**	**11.88**	**9.00**	**17.98**	**48.48**	**111.00**	**1352.16**
El Ferah area	F1	1.80	4.13	2.04	0.60	2.29	1.13	22.32	16.69	22.64	187.80
	F2	1.46	4.22	1.79	0.46	2.89	1.23	18.10	17.05	19.87	143.98
	F3	1.61	9.00	0.69	0.46	5.59	0.43	19.96	36.36	7.66	143.98
	F4	1.29	6.23	0.98	0.57	4.83	0.76	16.00	25.17	10.88	178.41
	ave	**1.54**	**5.90**	**1.38**	**0.52**	**3.90**	**0.89**	**19.10**	**23.82**	**15.26**	**163.54**
	min	**1.29**	**4.13**	**0.69**	**0.46**	**2.29**	**0.43**	**16.00**	**16.69**	**7.66**	**143.98**
	max	**1.80**	**9.00**	**2.04**	**0.60**	**5.59**	**1.23**	**22.32**	**36.36**	**22.64**	**187.80**
El Sahu I area	S1	37.81	1.50	81.05	0.01	0.04	2.14	468.84	6.06	899.66	3.13
	S2	62.90	1.46	80.86	0.40	0.03	1.29	779.96	5.90	897.55	125.20
	S3	53.36	1.52	80.92	0.35	0.03	1.52	661.66	6.14	898.21	109.55
	ave	**51.36**	**1.49**	**80.94**	**0.25**	**0.03**	**1.65**	**636.82**	**6.03**	**898.47**	**79.29**
	min	**37.81**	**1.46**	**80.86**	**0.01**	**0.03**	**1.29**	**468.84**	**5.90**	**897.55**	**3.13**
	max	**62.90**	**1.52**	**81.05**	**0.40**	**0.04**	**2.14**	**779.96**	**6.14**	**899.66**	**125.20**
El Sahu II area	S4	2.65	2.45	1.92	0.22	0.92	0.72	32.86	9.90	21.31	68.86
	S5	9.25	0.28	2.22	0.33	0.03	0.24	114.70	1.13	24.64	103.29
	S6	3.56	3.54	2.50	0.31	0.99	0.70	44.14	14.30	27.75	97.03
	ave	**5.15**	**2.09**	**2.21**	**0.29**	**0.65**	**0.55**	**63.90**	**8.44**	**24.57**	**89.73**
	min	**2.65**	**0.28**	**1.92**	**0.22**	**0.03**	**0.24**	**32.86**	**1.13**	**21.31**	**68.86**
	max	**9.25**	**3.54**	**2.50**	**0.33**	**0.99**	**0.72**	**114.70**	**14.30**	**27.75**	**103.29**

Significants values are in bold.

In the Himayer area, samples were analyzed for natural radioactivity concentrations, and the region was divided into Himayer I and Himayer II. In Himayer II, the mean values of ^226^Ra and ^40^K for each of the 21 analyzed samples were 71.89 and 690.98 Bq kg^−1^, respectively, higher than the respective world averages of 32 and 412 Bq kg^−1^ , Additionally, the mean values of ^238^U and ^232^Th were lower than the world averages of 33 and 45 Bq kg^−1^, respectively.

In the El Sahu area, samples were categorized as El Sahu I and El Sahu II. In El Sahu II, the average values of specific activities were 24.57 Bq kg^−1^ for ^226^Ra, 8.44 Bq kg^−1^ for ^232^Th, and 89.73 Bq kg^−1^ for ^40^K, all below the respective world averages. However, the average value of ^238^U was 63.90 Bq kg^−1^, higher than the world average of 33 Bq kg^−1^.

The results indicate areas with low radioactive deposits, such as the El Ferah area, El Sahu II area, and Himayer II area, and areas with high radioactive deposits, such as El Sahu I and Himayer I.

In El Sahu I, the highest average specific activities recorded were 636.82 Bq kg^−1^ for ^238^U and 898.47 Bq kg^−1^ for ^226^Ra, significantly exceeding the world averages. In contrast, the average values for ^232^Th and ^40^K were much lower at 6.03 Bq kg^−1^ and 79.29 Bq kg^−1^, respectively. The high ^238^U and ^226^Ra levels indicate a break in the uranium decay chain and a slight migration of uranium, as shown by the ^226^Ra/^238^U ratio of 1.65. This migration is due to the ease of uranium dissolution, while its daughter product ^226^Ra remains because the conditions for its dissolution are more difficult.

For Himayer I, the mean values for ^238^U and ^40^K were 16.24 Bq kg^−1^ and 203.45 Bq kg^−1^, respectively, both below the world averages. However, the mean values for ^232^Th and ^226^Ra were 83.83 Bq kg^−1^ and 732.60 Bq kg^−1^, respectively, both higher than the world averages.

In heavy mineral investigations, it was found that certain yellow ochre samples contain zircon, especially in Himayer I and El Sahu I areas, which likely contributes to their thorium and uranium content. Results from the Himayer I area show a disruption in the uranium decay chain, suggesting uranium migration due to its ease of dissolution, while its daughter product ^226^Ra remains due to more challenging dissolution conditions.

The mean measured activity concentrations of ^238^U, ^226^Ra, ^232^Th, and ^40^K in yellow oxide (commercial product materials) commonly used in wall pigments and dyes are presented in Table 2. The values are 20.61 Bq kg^−1^ for ^238^U, 16 Bq kg^−1^ for ^226^Ra, 11.94 Bq kg^−1^ for ^232^Th, and 137.72 Bq kg^−1^ for ^40^K, all below the respective world averages as established by UNSCEAR^37^.

**Table 2:** eU, eTh, Ra and K concentrations of the studied Commercial product.

	Sample	eU	eTh	eRa	K	Th/U	Ra/U	^238^U	^232^Th	^226^Ra	^40^k
	No	ppm	ppm	ppm	%			Bq kg^−1^	Bq kg^−1^	Bq kg^−1^	Bq kg^−1^
Commercial product	C1	0.76	2.28	1.75	0.01	3.00	2.30	9.42	9.21	19.43	3.13
	C2	4.38	3.55	1.50	0.40	0.81	0.34	54.31	14.34	16.65	125.20
	C3	1.05	0.35	2.89	0.02	0.33	2.75	13.02	1.41	32.08	6.26
	C4	1.99	6.70	1.22	0.10	3.37	0.61	24.68	27.07	13.54	31.30
	C5	4.08	3.42	1.55	0.76	0.84	0.38	50.59	13.82	17.21	237.88
	C6	2.61	3.74	0.45	0.26	1.43	0.17	32.36	15.11	5.00	81.38
	C7	2.79	1.65	0.95	0.42	0.59	0.34	34.60	6.67	10.55	131.46
	C8	1.00	1.99	0.74	0.55	1.99	0.74	12.40	8.04	8.21	172.15
	C9	1.23	2.14	2.39	0.83	1.74	1.94	15.25	8.65	26.53	259.79
	C10	1.77	3.18	0.19	0.43	1.80	0.11	21.95	12.85	2.11	134.59
	C11	1.01	0.74	1.69	0.30	0.73	1.67	12.52	2.99	18.76	93.90
	C12	1.33	2.05	2.18	0.64	1.54	1.64	16.49	8.28	24.20	200.32
	C13	1.77	3.25	0.05	1.18	1.84	0.03	21.95	13.13	0.56	369.34
	C14	1.04	6.23	1.00	0.17	5.99	0.96	12.90	25.17	11.10	53.21
	C15	1.44	2.54	2.13	0.25	1.76	1.48	17.86	10.26	23.64	78.25
	C16	0.95	3.01	1.45	0.66	3.17	1.53	11.78	12.16	16.10	206.58
	C17	0.20	3.40	1.29	0.50	17.00	6.45	2.48	13.74	14.32	156.50
	C18	0.52	2.98	2.53	0.44	5.73	4.87	6.45	12.04	28.08	137.72
	ave	**1.66**	**2.96**	**1.44**	**0.44**	**2.98**	**0.87**	**20.61**	**11.94**	**16.00**	**137.72**
	min	**0.20**	**0.35**	**0.05**	**0.01**	**0.33**	**0.25**	**2.48**	**1.41**	**0.56**	**3.13**
	max	**4.38**	**6.70**	**2.89**	**1.18**	**17.00**	**0.66**	**54.31**	**27.07**	**32.08**	**369.34**

Significants values are in bold.

**Fig. 9:**
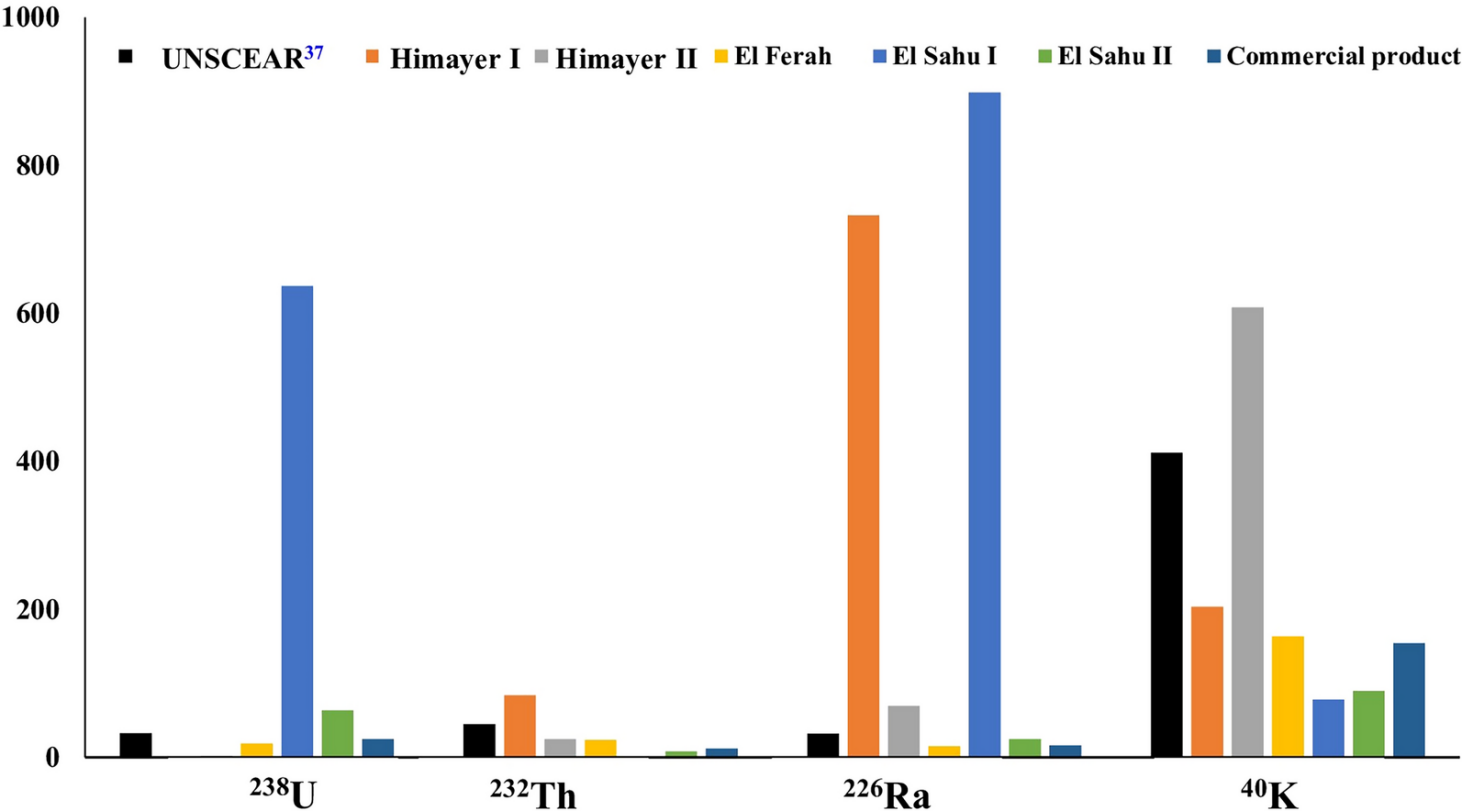
The average activities (Bq kg^−1^) for ^238^U, ^226^Ra, ^232^Th, and ^40^K for studies samples as well as the respective world averages set by UNSCEAR^37^.

**Fig. 10:**
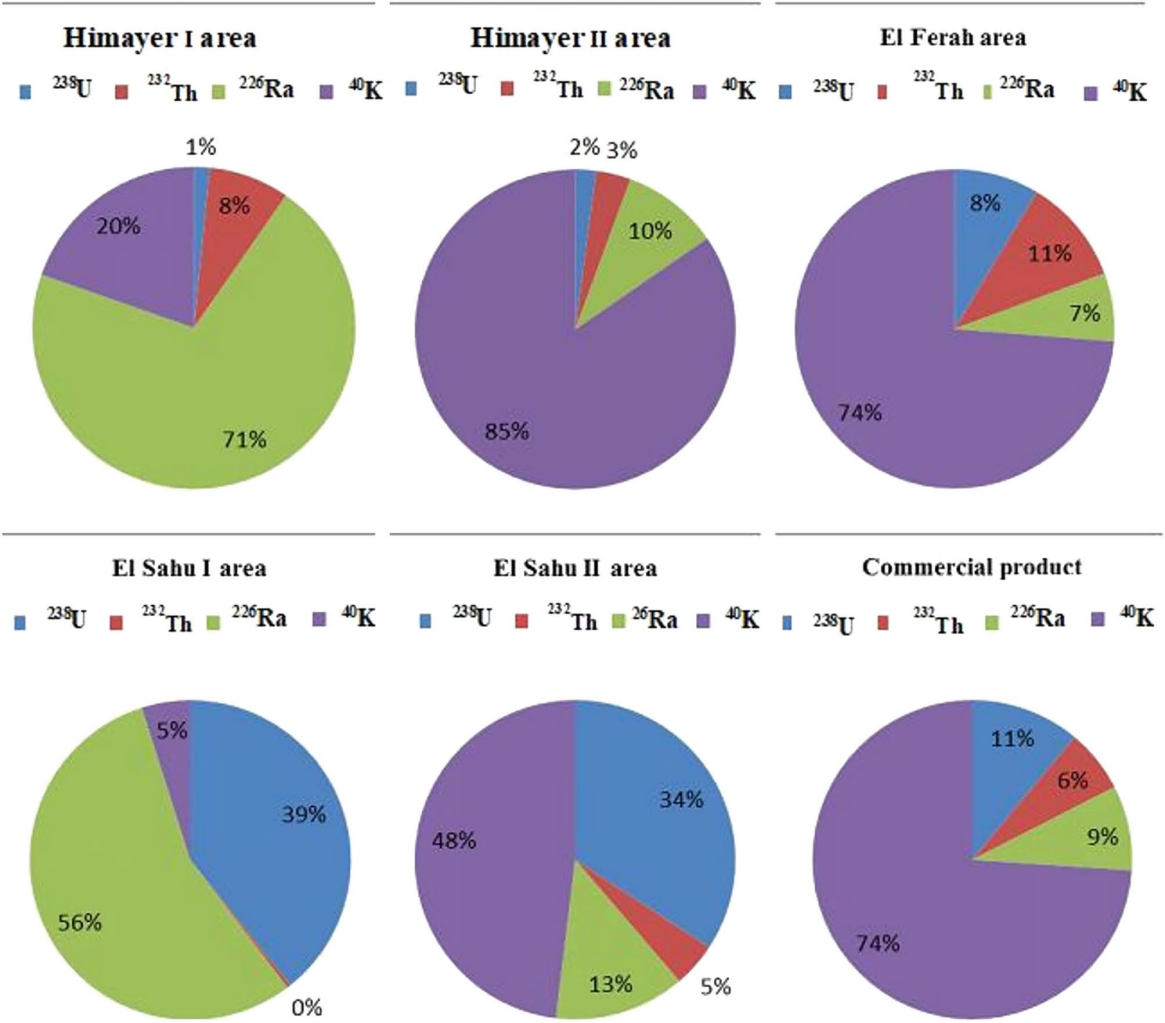
The average activities (%) for ^238^U, ^226^Ra, ^232^Th and ^40^K for the investigated samples.

Natural radioactivity concentrations of ^238^U, ^226^Ra, ^232^Th, and ^40^K in different samples were compared with world averages (Fig. 9). The contribution of these radionuclides in various localities is shown in Fig. 10.

The study reveals a significant variation in radionuclide contributions among the samples. In Himayer I and El Sahu I, ^226^Ra is the dominant radionuclide, contributing 71% and 56%, respectively. Conversely, ^40^K is the dominant radionuclide in Himayer II, El Ferah, El Sahu II, and commercial product samples, contributing 85%, 74%, 48%, and 74%, respectively.

### Th/U and Ra/U ratios indication in different locations of ochre sampling

The eTh/eU ratio serves as a valuable tool for characterizing geochemical facies, providing insights into oxidizing or reducing conditions. Under reducing conditions, uranium remains insoluble in its tetravalent oxidation state, while it becomes soluble and mobile in its hexavalent state. Thorium, on the other hand, typically remains in a single insoluble tetravalent state and is often geochemically associated with uranium. This ratio facilitates comparisons between different geochemical processes^38–40,49^.

In the studied locations of ochre, the eTh/eU ratio indicates uranium leaching and thorium accumulation in the Himayer I and II areas, suggesting oxidizing conditions. The high ratios (16.00 and 4.96, respectively) are associated with zircon mineralization. Conversely, El Sahu I has a very low eTh/eU ratio (0.03), indicating uranium deposition under reducing conditions. El Ferah, El Sahu II, and the commercial product have comparable uranium and thorium contents, likely due to adsorption on iron minerals under repeated oxidation–reduction conditions^40^.

The activity ratios of ^226^Ra/^238^U vary across the regions, reflecting the mobility of radium under different conditions. The ratios are as follows: Himayer I: 45.33–65.00 (average 51.50), Himayer II: 2.73–9.00 (average 5.73), El Ferah: 0.43–1.23 (average 0.89), El Sahu I: 1.29–2.14 (average 1.65), El Sahu II: 0.24–0.72 (average 0.55), Commercial product: 0.25–0.66 (average 0.87).

Radium typically exhibits intermediate mobility compared to uranium (IV) and uranium (VI). However, unlike uranium, radium tends to be less mobile in oxidizing conditions due to its strong adsorption by clay and iron minerals^41,42^, which are commonly found in oxidizing environments resulting from the weathering of host rocks^43,44^. ^226^Ra/^238^U average values that are lower than unity in El Ferah, El Sahu II, suggest the migration of uranium from the rocks.

The decrease in ^226^Ra solubility can be attributed to its co-precipitation with compounds such as (Ba, Pb) SO_4_^45^^.^ Saad^46^ Studies have shown that manganese ore deposits in the Um Bogma region are enriched in elements like lead, copper, zinc, and barium, suggesting that ^226^Ra may co-precipitate with lead and barium sulfates.

Additionally, investigations of dug or drilled wells in these rock formations have indicated elevated concentrations of chloride and sulfate ions (SO_4_^2−^) in underground water^47,48^^.^ These findings further support the presence of compounds that can influence the solubility and mobility of radium in the environment.

### Evaluation of radiological hazards

Radiation exposure from radionuclides encompasses both external sources, such as gamma radiation emitted by isotopes like ^238^U, ^232^Th, and ^40^K, internal sources, stemming from inhalation of ^222^Rn, ^220^Rn, and their short-lived progeny, which emit alpha particles^49^.

To assess associated radiation hazards, a comprehensive set of radiological parameters has been utilized (Table 3). These parameters, including Radium Equivalent Activity (Ra_eq_), External Hazard Index (H_ex_), Internal Hazard Index (H_in_), Absorbed Dose Rate (D), Annual Effective Dose (AED), and Excess Lifetime Cancer Risk (ELCR), were calculated for the studied materials and summarized in Tables 4 and 5. This table offers insights into potential radiological hazards and evaluates the risk of radiation exposure to human health.

**Table 3:** Important radiological parameters and indices.

Parameter	Definitions	The mathematical formula
Ra_eq_ (Bqkg^−1^)	Radium equivalent is a common index used to assess the radioactivity of Naturally Occurring Radioactive Materials (NORMs), specifically those containing varying amounts of ^226^Ra, ^232^Th, and ^40^K^34^. This index serves as a guideline for regulating safety standards in radiation protection for the general public in a given area	Ra_eq_(Bq kg^−1^) = ARa + 1.43ATh + 0.077AK ≤ 370^10,51– 54^
H_in_	Internal hazard index is used to assess the internal exposure caused by the inhalation of radon gas	H_in_ = (ARa/185) + (ATh/259) + (AK/4810) ≤ 1^53^
H_ex_	external hazard index is specifically designed to evaluate the outdoor or external radiation hazard to keep it at an insignificant level	H_ex_ = (ARa/370) + (ATh/259) + (AK/4810) ≤ 1^51–53^
D_out_ (nG h^−1^)	The outdoor external exposure originating from terrestrial radionuclides in soil was calculated in air at a height of 1 m above the ground surface, expressed in (nG h^-1^)	D_out_ (nGy h^−1^) = 0.462 * A_Ra_ + 0.604 * A_Th_ + 0.0417 * A_K_ ≤ 59^55,56^
D_in_ (nG h^−1^)	The indoor internal dose due to gamma-ray radiation, primarily influenced by materials in the building’s construction containing ^226^Ra, ^232^Th, and ^40^K, was computed in (nG h^-1^)	D_in_ (nGy h^−1^) = 0.92 · A_Ra_ + 1.1 · A_Th_ + 0.08 · A_K_ ≤ 84^57^
E_out_ (mSvy^−1^)	^The annual outdoor effective dose is determined based on the outdoor external dose rate (Dout) used to gauge radiation exposure levels over a fixed period of time (1 year)^	Eout = D_out_ × Occupancy Factor × Conversion Factor
		E_out_ = D_out_ (nGy h^-1^) × 1.226 × 10^–6^ mSv^10,58^
		Occupancy factor (20% of 8760 h in a year), and the conversion factor (0.7 Sv Gy^−1^) for converting absorbed dose in air to effective dose
E_in_	The annual indoor effective dose is the dose which a person receives in the indoor environment. The (Ein) depends on the indoor internal dose (Din) used to gauge radiation exposure levels over a fixed period of time (1 year)	Ein = D_in_ × Occupancy Factor × Conversion Factor
		Occupancy factor (80% of 8760 h in a year), and the conversion factor (0.7 Sv Gy^−1^) for converting absorbed dose in air to effective dose
		Ein = D_in_ (nGy h^-1^) × 4.905 × 10^–6^ mSv^10^
ELCR_out_	The radioactive factor used to determine whether gamma radiation exposure caused lethal cancer is called excess lifetime cancer	ELCRout = E_out_ × DL × RF ≤ 2.9 × 10^−3^^54,59–61^
		Here, E is the Annual Effective Dose Rate, DL is the average life duration (assuming 70 years), and RF is the fatal cancer risk per Sievert, assumed to be 0.05 for stochastic effects for the populace^62–65^

**Table 4:** Environmental hazard indices of samples from the different locations.

Area	Sample No	Ra.eq	H_ex_	H_in_	D_Out_	E_Out_	D_in_	E_hn_	ELCR*10^−3^
Himayir I area	H1	833.21	2.25	4.20	382.12	0.47	753.35	3.70	1.64
	H2	849.05	2.29	4.18	388.34	0.48	763.10	3.75	1.67
	H3	878.54	2.37	4.41	401.96	0.49	791.81	3.89	1.73
	H4	911.78	2.46	4.50	417.89	0.51	821.67	4.03	1.79
	Ave	868.14	2.35	4.33	397.58	0.49	782.48	3.84	1.71
	Min	833.21	2.25	4.18	382.12	0.47	753.35	3.70	1.64
	Max	911.78	2.46	4.50	417.89	0.51	821.67	4.03	1.79
	H5	191.45	0.52	0.79	92.29	0.11	179.23	0.88	0.40
	H6	116.39	0.31	0.40	54.88	0.07	104.50	0.51	0.24
Himayir II area	H7	111.59	0.30	0.42	52.08	0.06	99.75	0.49	0.22
	H8	154.47	0.42	0.57	70.98	0.09	135.19	0.66	0.30
	H9	180.07	0.49	0.70	85.83	0.11	165.38	0.81	0.37
	H10	110.67	0.30	0.45	53.45	0.07	103.70	0.51	0.23
	H11	171.89	0.46	0.67	82.78	0.10	160.00	0.79	0.36
	H12	204.95	0.55	0.82	99.60	0.12	193.25	0.95	0.43
	H13	121.04	0.33	0.51	57.50	0.07	111.60	0.55	0.25
	H14	137.18	0.37	0.55	65.55	0.08	126.81	0.62	0.28
	H15	120.81	0.33	0.51	58.06	0.07	112.92	0.55	0.25
	H16	195.50	0.53	0.77	92.62	0.11	178.53	0.88	0.40
	H17	86.76	0.23	0.32	40.21	0.05	76.83	0.38	0.17
	H18	124.41	0.34	0.52	58.63	0.07	113.54	0.56	0.25
	H19	215.08	0.58	0.88	104.21	0.13	202.46	0.99	0.45
	H20	182.96	0.49	0.67	88.96	0.11	171.26	0.84	0.38
	H21	177.44	0.48	0.66	88.04	0.11	170.19	0.84	0.38
	H22	174.12	0.47	0.74	85.66	0.11	167.47	0.82	0.37
	H23	143.70	0.39	0.57	69.77	0.09	135.14	0.66	0.30
	H24	155.25	0.42	0.60	74.65	0.09	144.03	0.71	0.32
	H25	221.35	0.60	0.87	109.86	0.13	213.41	1.05	0.47
	Ave	157.00	0.42	0.62	75.50	0.09	145.96	0.72	0.32
	Min	86.76	0.23	0.32	40.21	0.05	76.83	0.38	0.17
	Max	221.35	0.60	0.88	109.86	0.13	213.41	1.05	0.47
	F1	60.96	0.16	0.23	28.37	0.03	54.21	0.27	0.12
El Ferah area	F2	55.34	0.15	0.20	25.48	0.03	48.55	0.24	0.11
	F3	70.74	0.19	0.21	31.50	0.04	58.56	0.29	0.14
	F4	60.61	0.16	0.19	27.67	0.03	51.97	0.26	0.12
	ave	61.91	0.17	0.21	28.26	0.03	53.32	0.26	0.12
	min	55.34	0.15	0.19	25.48	0.03	48.55	0.24	0.11
	max	70.74	0.19	0.23	31.50	0.04	58.56	0.29	0.14
El Sahu I area	S1	908.56	2.46	4.89	419.43	0.51	834.60	4.10	1.80
	S2	915.62	2.47	4.90	423.45	0.52	842.25	4.13	1.82
	S3	915.43	2.47	4.90	423.25	0.52	841.87	4.13	1.82
	ave	913.20	2.47	4.90	422.04	0.52	839.57	4.12	1.81
	min	908.56	2.46	4.89	419.43	0.51	834.60	4.10	1.80
	max	915.62	2.47	4.90	423.45	0.52	842.25	4.13	1.82
El Sahu II area	S4	40.77	0.11	0.17	18.70	0.02	36.00	0.18	0.08
	S5	34.21	0.09	0.16	16.38	0.02	32.18	0.16	0.07
	S6	55.67	0.15	0.23	25.50	0.03	49.02	0.24	0.11
	ave	43.55	0.12	0.18	20.19	0.02	39.07	0.19	0.09
	min	34.21	0.09	0.16	16.38	0.02	32.18	0.16	0.07
	max	55.67	0.15	0.23	25.50	0.03	49.02	0.24	0.11
Recommended value or worldwide average		**370**	** ≤ 1**	** ≤ 1**	**59**	**0.07**	**84**	**0.41**	≤ 0.29*10^**–3**^

*D*_*Out*_ absorbed dose rate outdoor, *D*_*In*_ The absorbed Dose rate indoor, *D*_*Out*_ absorbed dose rate outdoor, *E*_*In*_ annual effective dose rate indoor.

**Table 5:** Environmental hazard indices of samples from yellow oxide product.

	Sample No	Ra.eq	H_ex_	H_in_	D_Out_	E_Out_	D_in_	E_hn_	ELCR*10^–3^
Comertial product	C1	32.84	0.09	0.14	14.67	0.02	28.25	0.14	0.06
	C2	46.8	0.13	0.17	21.58	0.03	41.11	0.2	0.09
	C3	34.58	0.09	0.18	15.94	0.02	31.57	0.15	0.07
	C4	54.66	0.15	0.18	23.91	0.03	44.74	0.22	0.1
	C5	55.28	0.15	0.2	26.21	0.03	50.06	0.25	0.11
	C6	32.87	0.09	0.1	14.83	0.02	27.73	0.14	0.06
	C7	30.2	0.08	0.11	14.38	0.02	27.55	0.14	0.06
	C8	32.97	0.09	0.11	15.83	0.02	30.17	0.15	0.07
	C9	58.9	0.16	0.23	28.31	0.03	54.7	0.27	0.12
	C10	30.84	0.08	0.09	14.35	0.02	26.84	0.13	0.06
	C11	30.26	0.08	0.13	14.39	0.02	28.06	0.14	0.06
	C12	51.47	0.14	0.2	24.54	0.03	47.4	0.23	0.11
	C13	47.77	0.13	0.13	23.59	0.03	44.5	0.22	0.1
	C14	51.19	0.14	0.17	22.55	0.03	42.15	0.21	0.1
	C15	44.34	0.12	0.18	20.38	0.03	39.3	0.19	0.09
	C16	49.39	0.13	0.18	23.4	0.03	44.71	0.22	0.1
	C17	46.01	0.12	0.16	21.44	0.03	40.8	0.2	0.09
	C18	55.9	0.15	0.23	25.99	0.03	50.1	0.25	0.11
	ave	**43.68**	**0.12**	**0.16**	**20.35**	**0.02**	**38.87**	**0.19**	**0.09**
	min	**30.2**	**0.08**	**0.09**	**14.35**	**0.02**	**26.84**	**0.13**	**0.06**
	max	**58.9**	**0.16**	**0.23**	**28.31**	**0.03**	**54.7**	**0.27**	**0.12**
Recommended value or worldwide average		**370**	** ≤ 1**	** ≤ 1**	**59**	**0.07**	**84**	**0.41**	≤ 0.29*10 ^**–3**^

*D*_*Out*_ absorbed dose rate outdoor, *D*_*In*_ The absorbed Dose rate indoor, *D*_*Out*_ absorbed dose rate outdoor, *E*_*In*_ annual effective dose rate indoor.

Significants values are in bold.

### Radium equivalent Ra_eq_ (Bq kg^−1^)

It is reassuring the observation of the radium equivalent activities (Ra_eq_) in the samples collected from El Ferah, El Sahu II, and Himayer II areas, as outlined in Table 4, range from 34.21 to 221.35 Bq kg^−1^, with a mean value of 87.49 Bq kg^−1^. Importantly, these values remain below the recommended maximum limit of 370 Bq kg^−1^. This limit corresponds to an annual effective dose of 1.5 mSv, according to guidelines established by NEA-OECD^50^. Therefore, based on the radium equivalent activity index, it can be inferred that the yellow ochre samples from these areas comply with the established safety standards for building materials.

Maintaining radium equivalent activities below the recommended maximum value is crucial in ensuring that the materials do not pose excessive radiation exposure risks to the public. The data from Tables 4 and 5 highlights significant differences in radium equivalent activity (Ra_eq_) among samples from different areas. Himayer I samples exhibit a Ra_eq_ ranging from 833.21 to 911.78 Bq kg^−1^, with an average of 868.14 Bq kg^−1^. Meanwhile, El Sahu I area samples show even higher Ra_eq_ values, ranging from 908.56 to 915.62 Bq kg^−1^, with an average of 913.20 Bq kg^−1^. Importantly, all samples from Himayer I and El Sahu I areas surpass the recommended limit of 370 Bq kg^−1^.

Conversely, the Ra_eq_ index values for yellow oxide (commercial product materials) used for painting range from 30.20 to 58.90 Bq kg^−1^, averaging at 43.68 Bq kg^−1^. Remarkably, these values are lower than the recommended maximum value for Ra_eq_, suggesting that the commercial product materials used for painting are safe for usage according to the study results.

### Internal and external hazard index (H_in_, H_ex_)

The external hazard indices (H_ex_) for yellow ocher samples from different areas were evaluated, with the following findings: For El Ferah samples, H_ex_ ranged from 0.15 to 0.19, with a mean value of 0.17. For El Sahu II samples, H_ex_ ranged from 0.09 to 0.15, with a mean value of 0.12. For Himayer II samples, H_ex_ varied from 0.23 to 0.60, with a mean value of 0.42. All mean H_ex_ values were found to be below the recommended threshold of unity (1). This indicates that the yellow ocher samples from El Ferah, El Sahu II, and Himayer II areas are not considered radioactive based on the external hazard index. Consequently, these samples can be deemed safe for usage in building purposes and other related applications.

Where the external hazard indices value yellow ocher samples varied from 2.25 to 2.46, with a mean value of 2.35 and 2.46 to 2.47 with mean value 2.46 for Himayer I and El Sahu I samples respectively. The external hazard indices values for Himayer I and El Sahu I yellow ocher samples exceeded the recommended threshold of unity, indicating radioactivity levels unsuitable for building purposes.

Finally, the external hazard indices values for yellow oxide used for painting were between 0.08 to 0.16 with an average 0.12. These values were below the recommended threshold, suggesting they can be safely used in construction.

Tables 4 and 5 outlines the minimum, maximum, and average values of the internal hazard index (H_in_) for the studied samples. In El Ferah, El Sahu II, and Himayer II areas, H_in_ ranged from 0.16 to 0.88, with a mean value of 0.34, all below the recommended maximum of one.

However, for Himayer I yellow ocher samples, H_in_ ranged between 4.18 and 4.50, averaging 4.33. El Sahu I area samples exhibited the highest H_in_ values, ranging from 4.89 to 4.90, with an average of 4.90, surpassing the recommended threshold.

On the other hand, H_in_ values for yellow oxide product materials used for painting ranged from 0.09 to 0.23, averaging 0.16, all below the recommended threshold of unity. Consequently, based on this study’s findings, these yellow oxide product materials can be deemed safe for usage.

#### Gamma radiation dose rate (DR)

When calculating absorbed dose rates from gamma radiation, the assumption is typically made that the contribution from other naturally occurring radionuclides, such as ^235^U, ^87^Rb, ^138^La, ^147^Sm, and ^178^Lu, is insignificant. This means that the analysis primarily focuses on the contributions from the more common radionuclides, namely ^226^Ra, ^232^Th, and ^40^K while disregarding the potential impact of the additional mentioned radionuclides.

The study analyzed gamma radiation dose rates across different regions, revealing that D_out_ values in El Ferah and El Sahu II were below the global average (59 nG h^−1^), averaging 28.26 and 20.19 nG h^−1^, respectively. Similarly, D_in_ values in these regions were also below the global average (84 nG h^−1^), with averages of 53.32 and 39.07 nG h^−1^, respectively. Consequently, D_tot_ values remained within permissible limits (≤ 143 nG h^−1^), averaging 81.58 and 59.26 nG h^−1^, respectively.

In contrast, in Himayer II, D_out_ averaged 75.5 nG h^−1^, D_in_ averaged 145.96 nG h^−1^, and D_tot_ averaged 221.46 nG h^−1^, all-surpassing global averages.

Himayer I and El Sahu I exhibited even higher D_tot_ values, approximately nine times higher than global averages. These elevated doses suggest a significant potential for increased radiation exposure in these regions, necessitating further investigation and consideration of local factors to assess associated health risks.

Conversely, D_out_ and D_in_ values for yellow oxide materials remained within recommended limits, averaging 20.35 and 38.87 nG h^−1^, respectively, with a D_tot_ average of 59.22 nG h^−1^, indicating negligible radioactive risk. Nonetheless, the study highlights the importance of using appropriate building materials, such as wood, to mitigate indoor exposure and align indoor exposures with outdoor levels^55^.

### Annual effective dose

The annual effective dose (E_in_) for yellow ocher samples from El Ferah and El Sahu II ranged from averaging 0.26 and 0.19 mSv y^−1^, respectively, which is lower than the global average of 0.41 mSv y^−1^ reported by UNSCEAR^10^. Consequently, the average total annual effective dose (E_out_ + E_i_n) for these samples is 0.29 and 0.21 mSv y^-1^, respectively, remaining below the global average of 0.52 mSv y^-1^ reported by UNSCEAR^10^.

In Himayer II, E_out_ and E_in_ values resulted in a total annual effective dose (0.09 + 0.72) of 0.81 mSv y^-1^, slightly higher than the global average (0.52 mSv y^-1^) but below the recommended limit for public exposure of 1 mSv y^−1^^66^.

However, Himayer I and El Sahu I exhibited significantly higher E_out_ and E_in_ values, with total annual effective doses (E_tot_) averaging 4.33 and 4.64 mSv y^−1^, respectively, approximately nine times higher than the world’s average.

For yellow oxide materials, E_out_ averaged 0.02 mSv y^−1^, E_in_ averaged 0.19 mSv y^−1^, and E_tot_ (E_in_ + E_out_) averaged 0.21 mSv y^−1^, all within the world’s average of 0.52 mSv y^−1^ reported by UNSCEAR^10^.

### Excess lifetime cancer risk (ELCR)

The Excess Lifetime Cancer Risk (ELCR) is a crucial metric for summarizing population risks , comparing pollutants and exposure routes. The recommended limit for ELCR is 3.75 × 10^−3^^67^, with a world average of 0.290 × 10^−3^^37,67^.

For outdoor exposure (ELCR_out_) from yellow ocher, Himayer I and El Sahu I exhibited significantly higher values, averaging 1.71 × 10^–3^ and 1.81 × 10^−3^, respectively, over six times higher than the world average. Conversely, Himayer II, El Ferah, and El Sahu II had ELCR_out_ values closer to the world average, ranging from 0.09 × 10^−3^ to 0.32 × 10^−3^. Yellow oxide (commercial product materials) showed even lower ELCR_out_ values, ranging from 0.06 × 10^−3^ to 0.12 × 10^−3^.

Based on these radiological risk parameters, yellow ocher samples from El Ferah and El Sahu II, as well as yellow oxide materials, are relatively free from radiological contamination and are recommended for building purposes. However, caution is advised when using yellow ocher from Himayer I and El Sahu I for construction purposes.

### Cluster analysis

**Fig. 11:**
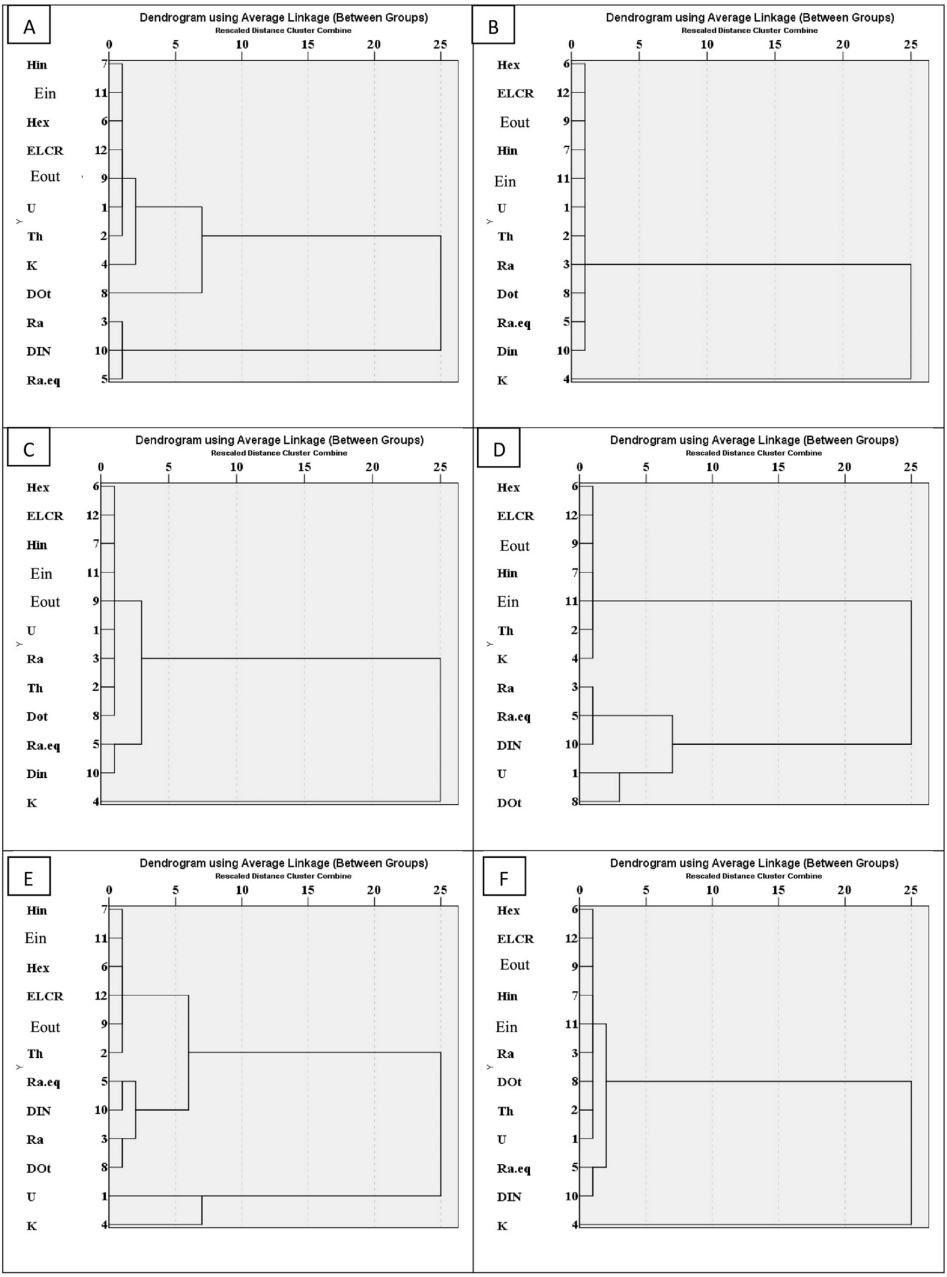
Dendrograms of the studied areas: (**a**) Himayer I area, (**b**) Himayer II area, (**c**) El Ferah area, (**d**) El Sahu I area, (**e**) El Sahu II area, and (**f**) Commercial product shows cluster of their radionuclide and their hazard indices.

The study utilized IBM SPSS Statistics version 25 for the multivariate statistical analysis, specifically employing cluster analysis with the average linkage method to establish relationships among various radiological variables. The resulting dendrogram from the Hierarchical Cluster Analysis (HCA) highlights these associations (Fig. 11).

In Himayer I, three significant clusters were identified: Cluster 1 comprises ^238^U, ^232^Th, and ^226^Ra along with pertinent environmental parameters, indicating their primary role in sediment radioactivity. Cluster 2 includes ^40^K, linked with E_out_.

For Himayer II and El Ferah, two clusters emerged: Cluster 1 encompasses ^238^U, ^232^Th, and ^226^Ra along with radiological hazard parameters, while Cluster 2 consists of ^40^K indicating its minimal contribution. In El Sahu I, Cluster I contains ^232^Th, ^226^Ra, and ^40^K, suggesting their role in sediment risk, while Cluster II comprises only ^238^U. In El Sahu II, clusters encompass ^232^Th, ^226^Ra, ^40^K, and ^238^U activity concentrations, each contributing to hazards. For Commercial Product (Yellow Oxide), Cluster One involves ^232^Th, ^226^Ra, and ^238^U, contributing to hazards, while ClusterII contains only ^40^K.

These cluster analyses offer insights into the primary contributors to radioactivity and environmental impact across different areas, facilitating a deeper understanding of the relationships between radiological variables.

## Conclusion

In summary, radiological studies were conducted on 35 yellow ochre raw material samples collected from El Ferah area, Wadi El Sahu area, and Himayer area in southwestern Sinai. Additionally, 18 representative yellow oxide samples (commercial product) derived from yellow ochre raw material were analyzed. XRD analysis revealed that the raw materials primarily consist of goethite, quartz and kaolinite in El Ferah area, hematite, kaolinite and quartz in Himayer area, and kaolinite, gypsum and quartz in Wadi El Sahu. The commercial product is mainly composed of goethite, quartz, and calcite. Heavy mineral investigation showed the presence of zircon and rare earth sulfate in some yellow ochre samples, possibly contributing to their thorium and uranium content. The specific activities of ^232^Th and ^226^Ra radionuclides in the Himayer I area were higher than the respective world averages, while in Himayer II, the averages of ^226^Ra and ^40^K radionuclides exceeded the respective world averages. In El Sahu I, the activities of ^238^U and ^226^Ra were higher than the world averages. The highest radioactivity in the studied samples is attributed to some yellow ochre samples containing zircon and rare earth sulfate, which may be responsible for their thorium and uranium content. El Ferah and El Sahu II had activities lower than the respective world averages for all studied radionuclides. There was notable variation in radionuclide contributions across samples, with ^226^Ra being the dominant contributor in Himayer I and El Sahu I. In the contrary, Himayer II, El Ferah, El Sahu II, and the commercial product were primarily influenced by ^40^K. The Th/U and Ra/U ratios exhibited significant changes, indicating dynamic physicochemical conditions during uranium leaching and deposition. Most radiological parameters in the ferruginous sediment and commercial product samples from El Ferah, El Sahu II, and Himayer II were within internationally recommended values. However, all radiological parameters observed in the Himayer I and El Sahu I samples were higher than the international recommended values. Hierarchical cluster analysis (HCA) identified key contributors to the hazards in different areas, emphasizing the significance of specific radionuclides in each location.

## Data Availability

All data generated or analyzed during this study are included in this published article.
